# Development of Novel Dengue NS1 Multiplex Lateral Flow Immunoassay to Differentiate Serotypes in Serum of Acute Phase Patients and Infected Mosquitoes

**DOI:** 10.3389/fimmu.2022.852452

**Published:** 2022-03-04

**Authors:** Szu-Chia Lai, Yu-Yine Huang, Jiunn-Jye Wey, Meng-Hung Tsai, Yi-Ling Chen, Pei-Yun Shu, Shu-Fen Chang, Yi-Jen Hung, Jiu-Nan Hou, Chang-Chi Lin

**Affiliations:** ^1^ Institute of Preventive Medicine, National Defense Medical Center, New Taipei City, Taiwan; ^2^ Center for Diagnostics and Vaccine Development, Centers for Disease Control, Ministry of Health and Welfare, Taipei City, Taiwan; ^3^ Diagnostic Device Group, Trison Technology Corporation, Taoyuan City, Taiwan; ^4^ Institute of Microbiology and Immunology, National Defense Medical Center, Taipei City, Taiwan

**Keywords:** dengue, serotype, NS1, lateral flow immunoassay, rapid test

## Abstract

Dengue is among the most rapidly spreading arboviral disease in the world. A low-cost, easy to use point-of-care diagnostic tool for the detection and differentiation of dengue virus serotypes could improve clinical management, disease prevention, epidemiological surveillance, and outbreak monitoring, particularly in regions where multiple serotypes co-circulate. Despite widespread deployment, no commercial dengue antigen diagnostic test has proven effective in differentiating among dengue virus serotypes. In the current study, we first established mAb pairs and developed a multiplex lateral flow immunoassay for the simultaneous detection of the dengue viral NS1 antigen and identification of serotype. The proposed system, called Dengue serotype NS1 Multiplex LFIA, provides high sensitivity and specificity. In testing for JEV, ZIKV, YFV, WNV, and CHIKV, the multiplex LFIA gave no indication of cross- reactivity with cell culture supernatants of other flaviviruses or chikungunya virus. In analyzing 187 samples from patients suspected of dengue infection, the detection sensitivity for serotype D1 to D4 was 90.0%, 88.24%, 82.61%, and 83.33% and serotype specificity was 98.74%, 96.13%, 99.39%, and 97.04%, respectively. Our multiplex LFIA can also identify mono- and co-infection of different serotype of dengue viruses in mosquitoes. The proposed Multiplex LFIA provides a simple tool for the rapid detection of dengue serotypes and in the differential diagnosis of fever patients in regions where medical resources are limited and/or multiple DENVs co-circulate.

## 1 Introduction

Dengue is the most common arboviral disease afflicting human beings. Dengue viruses are transmitted by mosquitoes of the genus *Aedes* (*Aedes aegypti* and *Aedes albopictus*). The disease is endemic in many tropical and subtropical regions (1–4), and has been observed expanding into new areas under the effects of climate change, land-use change, and urbanization (5–9). The four dengue virus serotypes are closely related and co-circulated globally (9, 10); however, they are distinct at the genetic and amino acid levels (11). The pathological outcome of severe dengue depends largely on the balance between viral factor and the genetic and immunological background of the host (3, 12). The factors associated with the risk of developing severe dengue include DENV infection with particular serotype, genotype, clade, or strain (13–15), the sequence of DENV serotype infections (13, 16), pre-existing intermediate anti-DENV antibodies titer (17, 18), and the timing between DENV infections (17, 19, 20). Dengue virus serotypes also differ in terms of virulence, disease severity, and epidemic capacity (15, 21–23). It appears that the genetic makeup of the host as well as their age, sex, and nutritional status also affect infection outcomes and disease severity (24). Infection with one serotype can confer lifelong homotypic immunity, but only maintains 2-3 months transient cross-protection against heterologous serotypes (17, 25). Cross-reactive antibodies or sub-neutralizing concentrations of antibodies binding to hetero-serotype dengue virus increase the risk of severe dengue disease (17, 18, 26). This mechanism can be explained by antibody-dependent enhancement (ADE), which is limited to a narrow range of pre-infection cross-reactive antibody titers (17).

Diagnosis is crucial to clinical management, prevention, and surveillance. In diagnosing dengue, clinicians cannot rely entirely on clinical manifestations, due to similarities with other acute febrile illnesses (3, 27). The selection of assay method depends largely on the stage of dengue infection. Within 0-5 day post-onset of symptoms (POS), dengue can be diagnosed *via* virus isolation, the detection of viral RNA using real-time RT-PCR, or the detection of viral antigens (e.g., NS1) *via* ELISA or rapid testing (28, 29). Molecular testing provides high sensitivity and specificity; however, molecular assays require a laboratory with specialized equipment, expensive reagents, a cold chain to maintain enzyme activity, and a trained operator to perform analysis (30–33). In dengue-endemic areas, limited molecular testing capacity can delay the acquisition of diagnostic results. Anti-DENV IgM antibodies can be detected at five days after fever onset, and anti-DENV IgG antibodies generally appear in low concentrations in cases of primary infection. However, in cases of secondary infection, anti-DENV IgG antibodies appear as early as three days post infection and titers rise rapidly after fever onset. The IgM/IgG ratio can be used to differentiate between primary and secondary infections. In dengue-endemic regions, antibody persistence from previous infection often makes it difficult to differentiate between earlier and current infections. Such cases require paired sera samples to detect seroconversion and confirm infection with dengue (30, 32, 33). Furthermore, serology assays cross react with a number of flaviviruses, such that they are unable to distinguish dengue virus serotypes and other flaviviruses (30, 34). Nonetheless, the low throughput of virus isolation, RNA detection and neutralization test renders this approach time-consuming, costly, and labor-intensive. The viral NS1 protein secreted from dengue virus-infected cells is present in early disease stages and can be detected in the blood for more than nine days after disease onset (30). NS1 testing requires only a single sample and eliminates the need for high-tech equipment. Note however that commercial ELISA and rapid tests vary widely in terms of sensitivity and specificity (30, 32, 33). The WHO TDR (Special Programme for Research and Training in Tropical Diseases) lists the benefits that a dengue test should ideally provide, including the ability to distinguish between dengue and other diseases with similar clinical presentations, high sensitivity, applicability during the acute phase of infection, rapid results, low-cost, ease of use, and stability at temperatures exceeding 30°C (30). At present, no commercial dengue NS1 point-of-care test kit is able to identify the serotypes of DENV-infection (28, 34–37); although, a number of systems with this function are currently in the development phase (38).

In a previous study, we reported an ELISA kit for the detection of dengue viral NS1 and the differentiation of dengue virus serotypes during the acute phase (39). In the current study, we used monoclonal antibodies to develop a point-of-care diagnostic assay based on immunochromatography, called the Dengue serotype NS1 Multiplex LFIA. The proposed system is inexpensive, user-friendly, and does not require sophisticated laboratory equipment. The multiplex LFIA detects the NS1 antigen of dengue virus and identifies the serotype by pairing a serotype-cross-reactive monoclonal antibody (mAb) with one of four serotype-specific mAbs. The Dengue serotype NS1 Multiplex LFIA developed in this study can be used to identify the specific dengue virus serotype causing the infection. The proposed system has also proven effective in distinguish the dengue virus from other flaviviruses and chikungunya viruses in clinical serum samples as well as in detecting instances of co-infection with two dengue virus serotypes in mosquitoes. This multiplex LFIA can be stored for a prolonged period in the field without the need for refrigeration. The proposed rapid test has completed the development and manufacturing stages of production. The proposed multiplex LFIA provides a useful tool for the epidemiological surveillance of circulating serotypes in acute dengue patients and infected mosquitoes, particularly in regions where medical resources are limited and/or multiple DENVs co-circulate.

## 2 Materials and Methods

### 2.1 Study Design

Our objective in this study was to develop a low-cost Dengue NS1 antigen LFIA for the detection of dengue virus and serotype identification. Applying the proposed DENV NS1 LFIA to a given serotype involved pairing one serotype-cross-reactive monoclonal antibody (conjugated using colloidal gold nanoparticles) with one of four serotype-specific monoclonal antibodies on an immobilized nitrocellulose membrane. Detection limits were defined using immunoaffinity-purified DENV NS1 proteins obtained from cell culture media of Vero cells infected with DENV1, 2, 3, or 4. Cross-reactivity was verified using cell culture supernatant from Vero cells infected with DENV1, 2, 3, or 4 or with Japanese encephalitis virus (JEV), Zika virus (ZIKV), West Nile virus (WNV), Yellow Fever 17D (YFV), or Chikungunya virus (CHIKV). Diagnostic performance was evaluated using clinical samples collected from suspected dengue-infected febrile patients and confirmed cases of dengue reported to the Centers for Disease Control, Taiwan. Serum samples were pre-validated using molecular, antibody, and/or dengue NS1 antigen ELISA tests. We compared the performance of DENV serotype NS1multiplex LFIA and reference tests in terms of sera identification. We also evaluated the feasibility of using DENV serotype NS1multiplex LFIA to detect and differentiate DENV serotypes from mosquitoes intrathoracically infected mono/or co-infected with DENVV1-4 or infected JEV, ZIKV, WNV, YFV, or CHIKV.

### 2.2 Virus

Eight dengue virus strains (DENV1 Hawaii, DENV2 16681, DENV3 H87, DENV4 H241, DENV1 8700828, DENV2 454009, DENV3 8700829, and DENV4 8700544), JEV SA14-14-2 strain, ZIKV ATCC VR 1843 strain, West Nile ATCC VR1510 strain, Yellow Fever 17D strain, and Chikungunya virus 0706aTw strain (Indonesia/0706aTw/2007/FJ807897) were propagated in Vero cells that had been incubated in RPMI 1640 medium containing 2% FBS at 37°C for 2 to 5 days. All viral titers were determined *via* plaque assays from Vero cells. NS1 proteins from the supernatant of Vero cells infected with DENV1-4, JEV, ZIKV, WNV, and YFV were then detected using Western blot analysis with anti-flavivirus mAb D_2_ 8-1 as the primary antibody, see **Supplementary Figure 1**.

### 2.3 Preparation of Monoclonal Antibodies

The hybridoma cells in this study were generated as described previously (39). Briefly, spleen cells from mice immunized with NS1 proteins of DENV1-4 were fused with NSI/1-Ab4-1 myeloma cells. Hybridoma cell lines that secreted specific antibodies against NS1 were identified *via* indirect ELISA using purified DENV NS1 as the coating antigen for each serotype. Positive hybridomas were cloned *via* limiting dilution. The mAbs were isotyped using a commercial mouse monoclonal antibody isotyping kit (IsoStripTM, Roche, Mannheim, Germany). Ascitic fluid was generated by intraperitoneally injecting pristine-primed mice with hybridomas. The mAbs were then purified from the ascitic fluid using a protein G-sepharose column (HiTrap protein G, GE Healthcare, Uppsala, Sweden) in accordance with the manufacturer’s instructions.

### 2.4 Antibody Selection for Lateral Flow Immunoassay

A total of 136 antibodies were harvested from hybridoma cell lines generated by immunizing mice with the viral NS1 antigen, as reported previously (39). The initial characterization of mAbs was performed *via* Western blot analysis of protein lysates from dengue virus-infected C6/36 cells as well as enzyme-linked immunosorbent assay (ELISA) involving individual serotype immunoaffinity-purified NS1 proteins. Candidate mAbs were tested both as capture and detection antibodies for all dengue virus serotypes using a standard capture ELISA procedure. Briefly, 96-well plates (Nunc Immuno Maxisorp, Thermo, Roskilde, Denmark) were coated with candidate mAbs and incubated overnight at 4°C. The wells were subsequently blocked using blocking buffer (PBS, 0.05% Tween, 5% skim milk) at 37°C for 1 h and then washed using wash buffer (PBS, 0.05% Tween). Viral culture supernatant or NS1 proteins were then serially diluted using blocking buffer, added to the wells, and incubated at 37°C for 1 h. The plates were washed before adding 100 μl of 0.8 μg/ml of potential mAbs-HRP to incubation at 37°C for 1 h. The microwell plates washed once again before adding 100 μl of TMB reagent followed by incubation at room temperature for 10 min. The reaction was stopped using 100 μl of 1 N sulfuric acid, whereupon the absorbance was read at 450 nm using a microplate auto reader. Serotype-specificity and limits of detection were assessed by testing each of the selected mAb combinations.

### 2.5 Development of DENV NS1 Multiplex LFIA for Serotype

Selected pairs of anti-DENV NS1 antibodies (mAb82-1.1 as a gold-labeled antibody as well as mAb51-1.1, 33-7.1, 43-1.3, and 22-1.5 as capture antibodies) were assembled as a strip plus cassette in LFIA format (Trison Technology Corporation, Taiwan, R.O.C).

#### 2.5.1 mAbs Conjugated to Colloidal Gold

Forty-nanometer colloidal gold nanoparticles were purchased from Tripod Nano Technology Corporation (Taiwan, R.O.C.). The mAb82-1.1-colloidal gold conjugates were prepared as follows. The antibodies were first diluted in a solution containing 2 mM Borax (pH 8.2). A mixture of 1 mL diluted mAb82-1.1 and 9 mL colloidal gold was incubated under gentle rotation (6 rpm) at room temperature for 20 min to allow the adsorption of mAbs onto the surface of the colloidal gold. To halt the coupling reaction, 1 mL of 10% BSA was added to the mAb82-1.1-colloidal gold mixture prior to incubation under gentle rotation (6 rpm) at room temperature over a period of 20 min. The unbound mAbs were then removed *via* centrifugation at 6,000 x g for 30 min. The supernatant was discarded and the resulting pellet was re-suspended in 1 mL of 2 mM borax (pH 8.2). This mixture was then centrifuged at 6,000 x g for 30 min. Following the removal of the supernatant, the pellet was resuspended in 1 mL of 0.1 M Tris-buffer (pH9.6) containing 22% sucrose, 2% BSA, 5% Trehalose and 1% casein, and then stored at 4°C.

#### 2.5.2 Assembly of DENV Serotype NS1 Multiplex LFIA

The immunochromatographic strip included four components: A sample pad (GL-b01, GL-b02), a conjugate pad (GL0194, Ahlstrom 8964, Ahlstrom 6613), a nitrocellulose membrane (Sartorius CN 140, PALL Vivid 170), and an adsorption pad (JY-X117). Goat anti-mouse IgG antibodies and four mouse anti-DENV specific antibodies (mAb51-1.1, 33-7.1, 43-1.3, 22-1.5) were separately applied to the nitrocellulose membrane for use as control and test lines, respectively. The nitrocellulose membrane was then dried at 40°C for 10 min to fix the antibodies. The mAb82-1.1-colloidal gold conjugate was sprayed onto the conjugate pad and then lyophilized with Freeze Dryer (FD12-5S; KINGMECH SCIENTIFIC CO., LTD., Taiwan). The condenser temperature was maintained at -60°C with the drying chamber maintained under vacuum of less than 10 Pa throughout the lyophilization process. The mAb82-1.1-colloidal gold conjugate pad was lyophilized for 8 h. The sample pad, pretreated conjugate pad, nitrocellulose membrane, and adsorption pad were pasted onto a backing card (300 mm×60 mm). Using a strip cutter, the resulting sheet was cut into 4 mm-wide strips, which were then assembled to form cassettes and stored under dry conditions until use.

#### 2.5.3 Test Procedure

LFIA implementation (i.e., manufacturer’s instructions) is outlined in the following (1). The test cassette and specimens were brought to room temperature (2). The test cassette was removed from the sealed foil pouch and placed on a flat and dry surface (3). 20 μL of running buffer (1% Trion X-100, 0.5% casein in 2mM borax) was added to the sample well. (4) 80 μL of specimen was added to the sample well and timing was begun. (5) The results were read by naked eye at 15 min. Results were not considered after 20 min.

### 2.6 Sensitivity Assay of DENV Serotype NS1 Multiplex LFIA

Sensitivity assays for antibody pairs involved measuring serially diluted immunoaffinity-purified DENV1-4 NS1 proteins (500, 250, 125, 62.5, 31.25, 15.625, and 7.812 ng/mL) for chromatographic presentation on the LFIA. The detection process was completed within 15 min, and the results were inspected visually.

### 2.7 Evaluating the Specificity of the DENV Serotype NS1 Multiplex LFIA Using Different Virus Culture Supernatants

Samples of cell culture supernatant from Vero cells infected with each virus (DENV1-4, JEV, ZIKV, WNV, YFV, and CHIKV) were used to confirm the specificity of the Dengue serotype NS1 multiplex LFIA *via* testing in triplicate. All virus titers ≧ 10^5 PFU/mL were tested using the LFIA in accordance with the manufacturer’s instructions.

### 2.8 Clinical Serum Samples and Laboratory Diagnosis

A total of 187 clinical serum samples were used for assessment. All clinical serum samples were collected during the acute phase (1-7 days after illness onset). In all cases of dengue detection in Taiwan, the Center for Disease Control (Taiwan CDC) must be notified including the submission of human serum samples. Taiwan has implemented a fever screening program at airports for the importing various infections including dengue fever. The another surveillance of dengue infection is based on a hospital-based reporting system tasked with notifying health authorities of all cases of dengue. The Taiwan CDC provided serum samples collected during 2016-2020, as follows: 91 dengue-confirmed serum samples, 5 Japanese encephalitis-confirmed serum samples, 3 Zika-confirmed serum samples, 5 chikungunya-confirmed serum samples, and 10 other febrile illness samples. The screening routine employed by the Taiwan CDC involves SYBR Green I-based quantitative one-step real-time multiplex RT-PCR assay for the differential diagnosis of various flaviviruses and chikungunya virus (40–42). In this study, DENV infection was defined as a febrile illness confirmed through the detection of DENV RNA *via* reverse transcription-polymerase chain reaction (RT-PCR), the detection of DENV NS1 antigens, and/or the detection of DENV-specific IgM/IgG enzyme-linked immunosorbent assay (43). The other 73 samples of suspected dengue infection used in this study were collected from three hospitals during 2016-2019 (Kaohsiung Armed Forces General Hospital, Zuoying Branch of Kaohsiung Armed Forces General Hospital, and Tangshan Branch of Kaohsiung Armed Forces General Hospital). The study protocol was approved by the Kaohsiung Armed Forces General Hospital Institutional Review Board (IRB no. KAFGH 104-048). One-step SYBR Green I-based real-time RT-PCR was performed using two sets of consensus primers, with one primer set targeting a region on the C gene to detect all flaviviruses and the other primer set targeting a region on the C gene to detect all DENV serotypes. The DENV serotypes of the positive results were then confirmed *via* DENV serotyping using four sets of serotype-specific primers targeting the C gene (40). DENV NS1 antigens were detected in clinical serum samples using the commercial Platelia Dengue NS1 AG ELISA kit (Bio-Rad, Marnes-la-Coquette, France) and/or ELISA for DENV NS1 serotyping (39). E/M-specific IgM and IgG capture enzyme-linked immunosorbent assays were used to detect DENV-specific IgM and IgG antibodies (43).

### 2.9 Using Dengue Serotype NS1 Multiplex LFIA to Detect DENV in Infected Mosquitoes

Colonized *Aedes aegypti* (Kaohsiung strain) were maintained under relative humidity of 80% at 28°C with a 16 h light/8 h dark cycle. Adults were provided 5% sucrose solution *ad libitum*. Laboratory mono-infection of *Aedes aegypti* was implemented as follows. One group of 5 female mosquitoes was intrathoracically inoculated with 0.1 μL of viral stock at a titer of (DENV1 8700828 strain: 5*10^6 PFU/mL, DENV2 454009 strain: 8*10^5 PFU/mL, DENV3 strain 8700829: 3*10^5 PFU/mL, DENV4 8700544 strain: 5*10^5 PFU/mL, JEV: 10^8 PFU/mL YF-17D 7*10^7 PFU/mL, WNV: 5.5*10^7 PFU/mL, CHIKV: 9*10^7 PFU/mL). Laboratory co-infection of *Aedes aegypti* was implemented as follows. One group of 5 female mosquitoes was intrathoracically inoculated with 0.1 μL of two equal-volume viral mixtures (D1+D2, D1+D3, D1+D4, D2+D3, D2+D4, and D3+D4). Five days after injection, mosquitoes were anesthetized and placed in 1.5 mL Eppendorf tubes sorted at -80°C. Each group of single mosquitoes was individually homogenized in 0.4 mL of PBS containing 1% NP40 for the detection of NS1 antigens using Dengue serotype NS1 Multiplex LFIA and the simultaneous extraction of viral RNA from the remaining lysate using QIAamp Viral RNA kit (Qiagen, Hilden, Germany) in accordance with the manufacturer’s instructions. cDNA was generated from RNA samples using the random primer by Superscript IV Reverse Transcriptase Kit (Thermo Fisher) at 50°C for 20 min and at 80°C for 10 min. PCR assays were performed in 50μl of reaction mixture containing 5μl of sample cDNA, 25μl of 2X PCR master mix (Thermo Fisher), and each of the specific primer pairs. PCR condition was as follow: 94°C for 2 min, then 30 cycles at 94°C for 30 s, 55°C for 30 s, 72°C for 30 s, and 72°C for 5 min. After amplification, a 10 μL aliquot of each product was analyzed *via* agarose gel electrophoresis. The sequence of primer pairs and the size of PCR-amplified DNA are listed in **Supplementary Tables 1** and **2**.

### 2.10 Statistical Analysis

The performance of the proposed dengue serotype NS1 multiplex LFIA in terms of serotyping sensitivity and specificity was compared with the summed results of RT-PCR, the dengue virus-specific IgM/IgG capture ELISA, and Dengue NS1 AG ELISA kit. The diagnostic accuracy, sensitivity, and specificity and the corresponding 95% confidence intervals (CI) for Dengue serotype NS1 Multiplex LFIA werwe determined using GraphPad Prism (version 7.0) software (GraphPad Software, San Diego, CA), where a *p* value of <0.05 indicated results of statistical significance.

## 3 Results

### 3.1 Antibody Selection for DENV Serotype NS1 Multiplex LFIA

In a previous study, 136 hybridoma cell lines were generated to produce anti-DENV NS1 monoclonal antibodies (39). Among the potential monoclonal antibodies, indirect ELISA, Western blot analysis, and dengue NS1 capture ELISA for serotype were used to select 10 antibodies, including 2 mAbs against DENV1-4 NS1 (serotype-cross), 2 mAbs against D1 NS1 (DENV1-specific), 2 mAbs against D2 NS1 (DENV2-specific), 2 mAbs against D3 NS1 (DENV3-specific), and 2 mAbs against D4 NS1 (DENV4-specific). The relative dengue NS1 capture ELISA for serotype values provided an initial assessment of detection performance in differential pairing. The DENV-group mAbs were used as capture antibodies for pairing with four serotype-specific mAbs. This selection was based on inter-serotype specificity and sensitivity. Finally, the following antibodies were selected to develop the multiplex LFIA: mAb 51-1.1 (DENV1-specific), mAb 33-7.1 (DENV2-specific), mAb 43-1.3 (DENV3-specific), mAb 22-1.5 (DENV4-specific), and mAb 82-1.1 (serotype-cross reactive). The five monoclonal antibodies underwent paired immunochromatographic analysis based on the serotype-cross reactive mAb conjugated with gold nanoparticles and four serotype-specific mAbs adsorbed on nitrocellulose membranes. The characteristics of the five mAbs are listed in **Table 1**.

**Table 1:** Characterization of reactions between mAbs and DENV NS1 proteins.

Hybridoma	Isotype	Type of epitope	Reactivity of four DENV serotypes (DENV1-4)									Specificity
			Western blot^a^					ELISA^b^				
			D1	D2	D3	D4		D1	D2	D3	D4	
82-1.1	IgG1,κ	linear	+	+	+	+		+	+	+	+	NS1
51-1.1	IgG2b,κ	conformational	+	–	–	–		+	–	–	–	NS1
33-7.1	IgG1,κ	conformational	–	+	–	–		–	+	–	–	NS1
43-1.3	IgG2a,κ	conformational	–	–	+	–		–	–	+	–	NS1
22-1.5	IgG1,κ	conformational	–	–	–	+		–	–	–	+	NS1

a The lysates of C6/36 cells infected with different dengue virus serotypes were treated using SDS-PAGE sample buffer and then subjected to gel electrophoresis before being transferred to a nitrocellulose membrane and blotted with each mAb.

b Different NS1 antigens were immunoaffinity-purified from cell culture supernatants of Vero cells infected with different serotypes of DENV. Microwell plates were coated with specific NS1 antigens and reacted with each mAb.

### 3.2 DENV Serotype NS1 Multiplex LFIA

**Figure 1:**
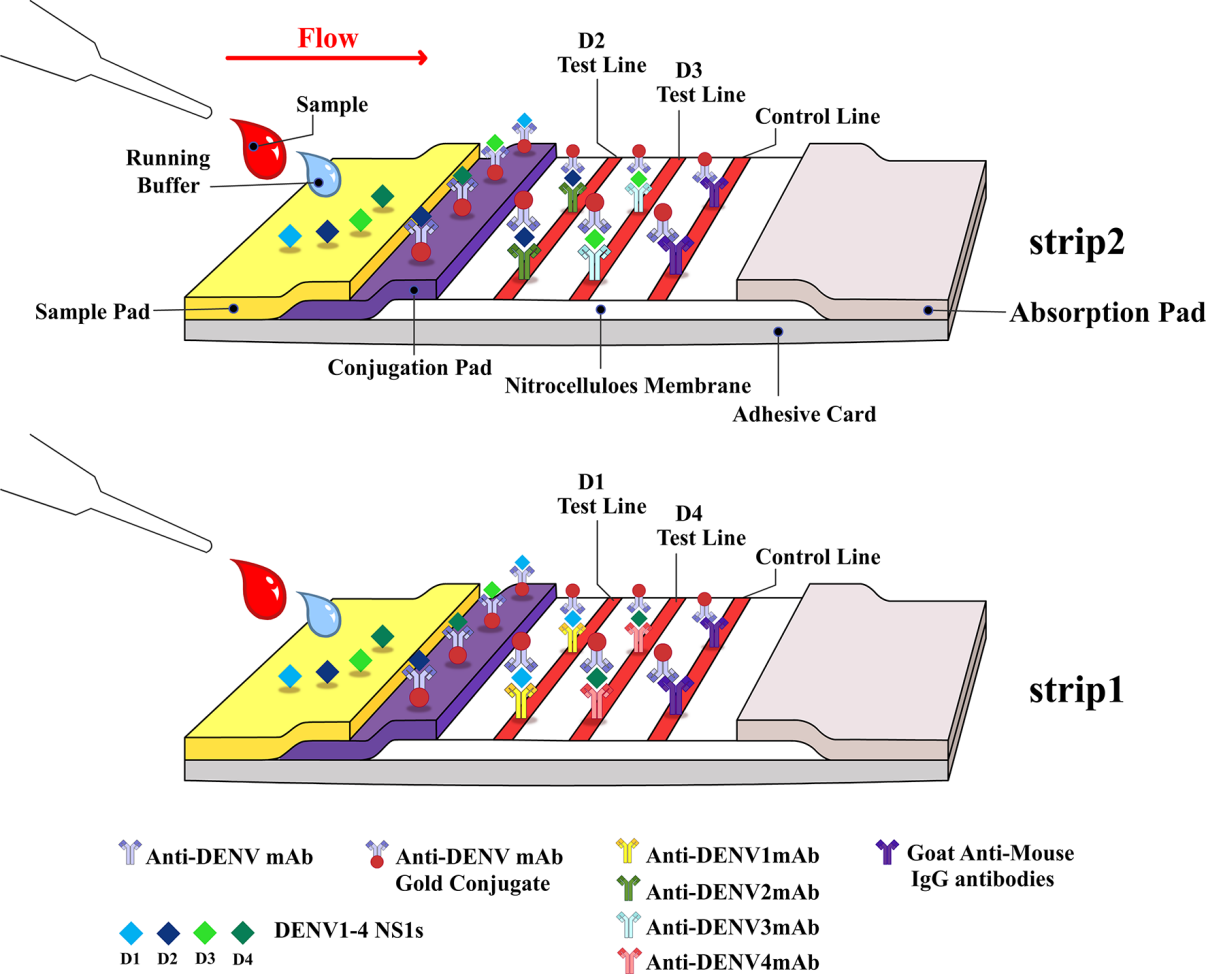
Schematic illustration showing Dengue serotype NS1 multiplex LFIA, comprising the following elements: Sample conjugation pad, membrane with immobilized antibodies, and absorption pad.

**Figure 2:**
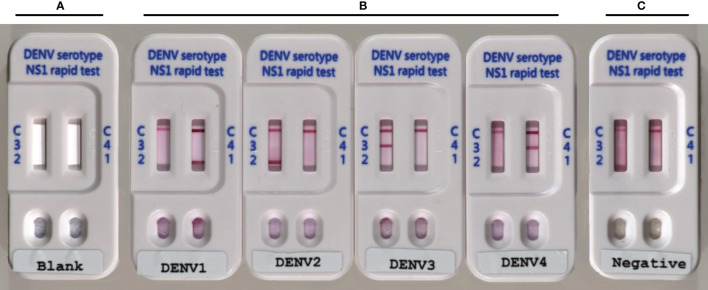
Photographs showing Dengue serotype NS1 multiplex LFIA: **(A)** Blank; **(B)** positive for DENV1-4 NS1 proteins, with colored band corresponding to the band at the test line; and **(C)** negative for DENV1-4, with bands at the test line (T) absent.

Our objective was to develop and validate a low-cost multiplex LFIA for the detection of DENV NS1 and the serotyping of dengue virus by pairing a serotype-cross-reactive monoclonal antibody (mAb82-1.1) with one of four serotype-specific mAbs (mAb 51-1.1, mAb 33-7.1, mAb 43-1.3, and mAb 22-1.5) to enable the detection of NS1 antigens and identification of DENV serotypes. We developed this device (Trison Technology Corporation Taiwan, R.O.C) to produce a multiplex LFIA using manufacture facilities that surpass national manufacturing standards. Serotype-cross-reactive mAb82-1.1 conjugated with colloidal gold and serotype-specific mAbs was immobilized on nitrocellulose membranes. Each strip contained two test lines. Strip one: DENV1-specific mAb 51-1.1 immobilized on T1 and DENV4-specific mAb 22-1.5 immobilized on T2; Strip two: DENV2-specific mAb 33-7.1 immobilized on T1 and DENV3-specific mAb 43-1.3 immobilized on T2, as shown in **Figure 1**. Anti-mouse IgG secondary antibodies were immobilized on the C-line for the capture of colloidal gold - mAb82-1.1-NS1 complex and colloidal gold -mAb82-1.1 conjugates. Images showing the design of the Dengue serotype NS1 multiplex LFIA device are presented in **Figure 2A**. When specimens containing the NS1 antigen were placed in the well, the DENV1-4 NS1 antigen reacted with the mAb82-1.1-colloidal gold to form NS1 antigen-antibody-colloidal gold complex. The complex was then captured by immobilized dengue serotype-specific mAbs. We performed chromatographic analysis on the multiplex LFIA using cell culture supernatants from Vero cells infected with DENV1-4. DENV *serotype 1* positive result: Both the control line and D1 test line appear, and the remaining test lines are invisible; DENV *serotype 2* positive result: Both the control line and D2 test line appear, and the remaining test lines are invisible; DENV *serotype 3* positive result: Both the control line and D3 test line appear, and the remaining test lines are invisible; DENV *serotype 4* positive result: Both the control line and D4 test line appear, and the remaining test lines are invisible. Images of the above results are presented in **Figure 2B**. Negative: Only control lines appear; i.e., the absence of D1 to D4 lines indicates negative results (**Figure 2C**). Invalid: No control line appears, thereby necessitating re-testing using a new test kit.

### 3.3 Analytic Sensitivity

The detection limit for each antibody pair was defined as the value 2-fold higher than the lowest concentration of DENV serotype NS1 multiplex LFIA that produced no visible color at T lines (D1-D4 test lines), as viewed by the naked eye. Immunoaffinity-purified DENV NS1 proteins were diluted serially and assayed using the multiplex LFIA in accordance with the manufacturer’s instructions. Analytical sensitivity was assessed using 2-fold serial dilutions of immunoaffinity-purified DENV NS1 proteins of DENV1, 2, 3, and 4 (at 1,000, 500, 250, 125, 62.5, 31.25, and 15.625 ng/mL). The 100% detection endpoints were 31.25 ng/mL for DENV1, DENV2, and DENV4. For DENV3, the 100% detection endpoint was 15.625 ng/mL (**Table 2** and **Supplementary Figure 2**). Thus, our results indicate that the limits of detection for the DENV 1-4 NS1 proteins ranged from 15.625 to 31.25 ng/mL.

**Table 2:** Detection limits of DENV serotype NS1 multiplex LFIA.

NS1 detection assay	DENV1	DENV2	DENV3	DENV4
	31.25 ng/mL	31.25 ng/mL	15.625 ng/mL	31.25 ng/mL

Sensitivity of DENV serotype NS1 multiplex LFIA in the detection of NS1. Each NS1 protein serotype was immunoaffinity-purified and serially diluted prior to analysis.

### 3.4 Analytic Specificity

Cross-reactivity performance was evaluated using NS1 proteins released from flavivirus-infected Vero cells into the cell culture supernatant. Vero cells were infected individually with the four DENV serotypes (DENV1, 2, 3, and 4) or with four other flaviviruses (ZIKV, JEV, WNV, and YFV) or CHIKV. Cell culture supernatant was collected and tested in triplicate using the DENV serotype NS1 multiplex LFIA. The final test concentrations of viruses are listed in **Table 3**. The data in **Table 3** indicate that specific DENV signals for each DENV serotype were observed on the strip, with no detectable mutual cross-reactivity. Note that none of the assays presented cross-reactivity with supernatant containing NS1 proteins from Japanese encephalitis virus, Zika virus, West Nile virus, Yellow fever virus, or Chikungunya virus particles. Furthermore, none of the DENV serotyping test lines reacted with the other non-targeted dengue virus serotypes, indicating that the assay has excellent specificity for DENV serotypes (**Table 3** and **Supplementary Figure 3**).

**Table 3:** Cross-Reactivity (Analytic Specificity).

Virus	Test concentration	Reactivity of a virus as a function of the number of assays with positive result/total number of assays					
		Dengue NS1 Ag (SD)	Dengue NS1 Ag strip (Bio-Rad)	DENV serotype NS1 multiple LFIA			
				D1	D2	D3	D4
Dengue virus-1	2*10^6 pfu/ml	3/3	3/3	**3/3**	0/3	0/3	0/3
Dengue virus-2	10^6 pfu/ml	3/3	3/3	0/3	**3/3**	0/3	0/3
Dengue virus-3	8*10^ 5pfu/ml	3/3	3/3	0/3	0/3	**3/3**	0/3
Dengue virus-4	5*10^5 pfu/ml	3/3	3/3	0/3	0/3	0/3	**3/3**
Japanese encephalitis virus	10^7pfu/ml	3/3	0/3	0/3	0/3	0/3	0/3
Zika virus	10^7 pfu/ml	3/3	0/3	0/3	0/3	0/3	0/3
Yellow Fever 17D	2*10^5 pfu/ml	0/3	0/3	0/3	0/3	0/3	0/3
West Nile virus	5*10^7 pfu/ml	3/3	0/3	0/3	0/3	0/3	0/3
Chikungunya virus	5*10^7 pfu/ml	0/3	0/3	0/3	0/3	0/3	0/3

In bold: Only to highlight the assay results.

We also compared two commercial DENV rapid tests. Standard Diagnostics DENV rapid tests and Bio-Rad DENV NS1 Ag strips using virus-infected cell culture supernatant from Vero cells. The Standard Diagnostics test revealed cross-reactivity with ZIKV, JEV, and WNV. The Bio-Rad test demonstrated specificity with DENV and no cross-reactivity with non-DENV flaviviruses. Note that neither commercial kit is able to identify the serotype of dengue virus (**Table 3**).

### 3.5 Validation Using Clinical Serum Samples

#### 3.5.1 Characterization

The clinical performance of the DENV serotype NS1 multiplex LFIA was evaluated by collecting 187 serum samples that met the criteria as a suspected case of acute DENV during the period from 2016 to 2020. The frozen sera samples were banked before being used in the rapid tests. The criteria for confirmation as a case of dengue included positive detection of RNA, antigens, or antibodies *via* laboratory diagnoses. In this study, the clinical serum samples were pre-validated using the molecular test for dengue virus infection using dengue serotype-specific multiplex one-step SYBR green I-based real-time RT-PCR (40). Patient blood samples were also tested for antibodies using DENV-specific capture IgM/IgG ELISA (43) as well as the Platelia Dengue NS1 Ag ELISA kit (Bio-Rad, Marnes-la-Coquette, France) or ELISA for DENV NS1 serotyping (39) for the detection of NS1 antigens of DENV. The results are shown in **Table 4**. Reference tests among 187 cases produced positive results for dengue virus in 104 samples following RT-PCR, 17 positive results for dengue virus following specific IgM/IgG capture ELISA, and 98 positive results for dengue virus following NS1 antigen capture ELISA (see **Table 4**). Among the 104 dengue virus RT-PCR-positive samples, we identified 30 cases of DENV1 infection, 33 cases of DENV2 infection, 23 cases of DENV3 infection, and 18 cases of DENV4 infection. In addition, 1 sample that produced negative RT-PCR results then tested positive following analysis using dengue virus-specific IgM/IgG capture ELISA and Dengue NS1 Ag ELISA. Thus, we confirmed 105 cases of dengue virus infection. The additional 82 samples of non-dengue viral infection tested negative when using serotyping RT-PCR, dengue virus-specific IgM/IgG capture ELISA, and Dengue NS1 Ag ELISA. The 82 cases of non-dengue virus included 5 cases of JEV-infection, 3 cases of ZIKV-infection, 5 cases of CHIKV-infection, and 69 cases involving other inflammation with fever. **Table 5-1** lists background information of the population from which clinical serum samples were obtained. **Table 5-2** details the clinical serum samples validated using serotyping RT-PCR, IgM/IgG capture ELISA, Dengue NS1 Ag ELISA (using Platelia Ag ELISA or our serotyping ELISA), and dengue serotype NS1 multiplex LFIA, and JEV, ZIKV, CHIKV were confirmed by RT-PCR methods. Testing results were further analyzed to evaluate the clinical performance of Dengue serotype NS1 multiplex LIFA in terms of sensitivity and specificity.

**Table 4:** Detection results of RT-PCR, Dengue NS1 antigen ELISA, and Dengue specific IgM/IgG capture ELISA when applied to 187 serum samples.

Reference tests	N	Percentage (%)
RT-PCR+/NS1-/IgM/IgG-	7	3.74
RT-PCR+/NS1+/IgM/IgG-	81	43.32
RT-PCR+/NS1+/IgM/IgG+	16	8.56
RT-PCR-/NS1+/IgM/IgG+	1	0.53
RT-PCR-/NS1-/IgM/IgG-	82	43.85
Total	187	100.00

Result composition for 187 cases using three reference methods: Dengue serotype-specific 1-step SYBR Green I-based RT-PCR, Platelia Dengue NS1 AG ELISA/or Dengue NS1 ELISA for serotype, and dengue virus specific IgM/IgG capture ELISA.

**Table 5-1:** Summary of clinical serum samples in this study.

Sample group	(n=)	Male : Female	Median age, years (range)	Original infection	(n=)
DENV1	30	16:14	35 (20-60)	Taiwan	(4)
				Cambodia	(3)
				Indonesia	(7)
				Laos	(1)
				Malaysia	(8)
				Thailand	(3)
				Vietnam	(3)
				Unknown	(1)
DENV2	34	19:15	46 (8-73)	Taiwan	(11)
				Cambodia	(2)
				India	(1)
				Indonesia	(10)
				Philippines	(1)
				Singapore	(1)
				Thailand	(2)
				Vietnam	(5)
				Unknown	(1)
DENV3	23	13:10	31 (8-43)	Indonesia	(11)
				Malaysia	(1)
				Myanmar	(2)
				Philippines	(8)
				Singapore	(1)
DENV4	18	9:9	38.5 (18-62)	Indonesia	(7)
				Myanmar	(1)
				Philippines	(1)
				Singapore	(2)
				Thailand	(1)
				Vietnam	(6)
Other fever	82	49:33	34.5 (14-73)	Taiwan	(75)
				Maldives	(2)
				Myanmar	(3)
				Thailand	(2)

**Table 5-2:** Description of serum samples (n=187) used in evaluating the performance of DENV serotype NS1 multiplex LFIA according to the DENV serotype, JEV, ZIKV, and CHIKV as well as days after fever onset.

Days after fever onset	Dengue group					JEV	ZIKV	CHIKV	Unknown fever	Number of positive using DENV serotype NS1 multiplex LFIA			
	DENV1	DENV2	DENV3	DENV4	total					D1	D2	D3	D4
1	7	4	5	5	21	0	1	2	17	6	6	4	6
2	7	10	8	4	29	0	0	1	14	6	7	6	2
3	4	6	4	2	16	1	1	2	12	5	6	5	3
4	4	6	5	5	20	0	0	0	10	3	7	4	6
5	0	4	0	1	5	0	0	0	8	0	3	0	1
6	3	2	1	0	6	2	0	0	6	3	2	1	0
7	0	1	0	0	1	0	0	0	1	0	1	0	0
8	1	0	0	0	1	1	1	0	0	2	1	0	0
9	0	0	0	0	0	0	0	0	0	0	0	0	0
10	0	0	0	0	0	1	0	0	0	0	0	0	1
unknown	4	1	0	1	6	0	0	0	1	4	3	0	1
total	30	34	23	18	105	5	3	5	69	29	36	20	20

#### 3.5.2 Sensitivity and Specificity of LFIA Strips for Serotype Using Clinical Serum Samples

A total of 187 serum samples were used to assess the sensitivity and specificity of DENV serotype NS1 multiplex LFIA, the results are presented in **Table 6**. The serotype sensitivity of the LFIA strip was defined as the number of measured true DENV serotype positives that had been pre-validated positive for dengue virus by reference methods, including molecular RT-PCR, dengue virus-specific IgM/IgG capture ELISA, and Dengue NS1 Ag ELISA. Sensitivity analysis was performed on 105 serum samples of dengue infection (including 104 serum samples confirmed as positive using RT-PCR, and 1 serum sample that tested negative using dengue-specific RT-PCR but positive using dengue-specific IgM/IgG capture ELISA and Dengue NS1 Ag ELISA). The sensitivity of the DENV serotype NS1 Multiplex LFIA was as follows: 27/30 (90%) for D1 test, 30/34 (88.24%) for D2 test, 19/23 (82.61%) for D3 test, and 15/18 (83.33%) for D4 test (see **Tables 6** and **7**).

**Table 6:** Performance of DENV serotype NS1 multiplex LFIA in detection of NS1 in acute-phase sera.

Virus	Reference test	Total number of serum samples (n=187)		Number of serum samples that tested positive using DENV serotype NS1 multiplex LFIA				
				D1 test line	D2 test line	D3 test line	D4 test line	Overall
DENV1^a^	Serotype RT-PCR	30	total DENVs: 105	27	2	0	0	total: 91
DENV2^a^	Serotype RT-PCR	33		1^g^	29	1^g^	1^g^	
DENV3^a^	Serotype RT-PCR	23		0	0	19	1	
DENV4^a^	Serotype RT-PCR	18		0	0	0	15	
DENVs^b^	NS1 AG ELISA/Dengue IgM/IgG capture ELISA	1		0	1	0	0	
ZIKV^c^	RT-PCR/sequence	3		0	0	0	0	0
JEV^d^	RT-PCR	5		1^h^	1^h^	0	0	1^h^
CHIKV^e^	RT-PCR	5		0	0	0	0	0
Unknown^f^	RT-PCR/ELISA/Dengue IgM/IgG capture ELISA/NS1 AG	69		0	3^i^	0	3^i^	4^i^

a A positive result was obtained using Dengue serotype-specific 1-step SYBR Green I-based real-time RT-PCR.

b A negative result was obtained using Dengue serotype-specific 1-step SYBR Green I-based real-time RT-PCR, whereas a positive result was obtained using Platelia Dengue NS1 AG ELISA, Dengue NS1 ELISA for serotype, and dengue virus specific IgM/IgG capture ELISA.

c A positive result was obtained using Flavivirus 1-step SYBR Green I-based real-time RT-PCR and sequences identified as Zika virus.

d A positive result was obtained using JEV specific 1-step SYBR Green I-based real-time RT-PCR.

e A positive result was obtained using CHIKV specific 1-step SYBR Green I-based real-time RT-PCR.

f A negative result was obtained using flavivirus and alphavirus 1-step SYBR Green I-based real-time RT-PCR, flavivirus and alphavirus specific IgM/IgG capture ELISA, Platelia Dengue NS1 AG ELISA, and/or Dengue NS1 ELISA for serotype.

g D1, D3, and D4 test lines presenting cross-reactivity with DENV2.

h D1 and D2 test lines presenting weak cross-reactivity with JEV.

i Four serum samples from unknown fever, two of which presented cross-reactivity with D2 and D4 test lines at the same time.

**Table 7:** Serotype specificity and sensitivity of DENV serotype NS1 multiplex LFIA for detection of NS1 in acute-phase sera (n=187).

DENV serotype NS1 multiplex LFIA	Number of serum samples with the following results:				Serotype sensitivity [%(95%CI)]	Serotype specificity [%(95%CI)]	Accuracy [%(95%CI)]
	True positives	True negatives	False positives	False negatives			
DENV1 Test line	27	155	2	3	90.00 (73.47-97.89)	98.74 (95.53-99.85)	97.35 (93.93-99.14)
DENV2 Test line	30	147	6	4	88.24 (72.55-96.70)	96.13 (91.77-98.57)	94.71 (90.49-97.43)
DENV3 Test line	19	163	1	4	82.61 (61.22-95.05)	99.39 (96.65-99.98)	97.33 (93.87-99.13)
DENV4 Test line	15	164	5	3	83.33 (58.58- 96.42)	97.04 (93.23- 99.03)	95.72 (91.74- 98.14)

Dengue serotype NS1 multiplex LFIA was compared with RT-PCR reference method. PPV, positive predictive value; NPV, negative predictive value; CI, confidence interval.

Specificity was defined as the number of true negatives divided by the number of samples confirmed negative by dengue specific RT-PCR, dengue specific IgM/IgG capture ELISA, and dengue NS1 antigen capture ELISA. Serum samples were used to calculate specificity of the DENV serotype NS1 multiplex LFIA for each serotype. Among the clinical testing, one of the DENV2 positive serum samples reacted to all D1-D4 test lines (D2) and showed a strong visible test line, while the other test lines showed weak visible color (data not shown). **Table 6** lists the number of dengue virus false positives for each serotype test line, as follows: One JEV sample little cross-reacted with D1 and D2 test lines (very weak visible color) and two serum samples from unknown fever cross-reacted with both D2 and D4 test lines (**Table 6**). Thus, serotype specificity was as follows: 155/157 (98.74%) for D1 test, 147/153 (96.13%) for D2 test, 163/164 (99.39%) for D3 test, and 164/169 (97.04%) for D4 test (**Table 7**). Serotype accuracy was 97.35%, 94.71%, 97.33%, and 95.72% for D1 to D4 test, respectively (**Table 7**).

**Table 8** illustrates the overall diagnostic performances of DENV serotype NS1 multiplex LFIA, when applied to 187 clinical serum samples, including 105 samples from patients with dengue viral infections and 82 samples from patients with non-dengue viral infections. Thus, the diagnostic accuracy of DENV serotype NS1 multiplex LIFA was 89.84% (95% CI, 84.59 to 93.77%), with specificity of 93.90% (77/82) (95% CI, 86.34 to 97.99%), sensitivity of 86.67% (91/105) (95% CI, 78.64 to 92.51%), positive predictive value of 94.97% (95% CI, 88.58% to 97.71%), and negative predictive value of 84.62% (95% CI, 77.10 to 89.98%).

**Table 8:** Overall diagnostic accuracy and sensitivity of DENV serotype NS1 multiplex LFIA.

Total number of serum samples	Dengue positive^a^	DENV serotype NS1 multiplex LFIA								
		True positives	True negatives	False positives	False negatives	Sensitivity [%(95%CI)]	Specificity [%(95%CI)]	Accuracy [%(95%CI)]	PPV [%(95%CI)]	NPV [%(95%CI)]
187	105	91	77	5	14	86.67 (78.64-92.51)	93.90 (86.34-97.99)	89.84 (84.59-93.77)	94.79 (88.58-97.71)	84.62 (77.10-89.98)

a A positive result was obtained using Dengue serotype-specific 1-step SYBR Green I-based real-time RT-PCR, Dengue NS1 Ag ELISA, and/or dengue virus specific IgM/IgG capture ELISA.

### 3.6 Validation of DENV Serotype NS1 Multiplex LFIA Using Mosquitoes Infected With DENV, JEV, ZIKV, WNV, YFV, and CHIKV

In Taiwan, nearly all dengue-infections involve a single serotype; i.e., it is difficult to find patients co-infected with multiple serotypes. We therefore assessed the performance of Dengue serotype NS1 Multiplex LFIA when applied to mosquitoes infected simultaneously with two different serotypes. We evaluated the ability of the Dengue NS1 multiplex LFIA to detect NS1 protein and identifying the serotype of dengue virus from a single infected mosquito. The detection of the NS1 antigen was evaluated using mono-infection or co-infection, wherein *Aedes aegypti* underwent direct intrathoracic microinjection respectively with one serotype (DENV1~4) or co-injection with two serotypes (DENV1/DENV2, DENV1/DENV3, DENV1/DENV4, DENV2/DENV3, DENV2/DENV4, and DENV3/DENV4). We also evaluated the specificity of the LFIA on JEV, ZIKV, WNV, YFV, and CHIKV infected *Aedes aegypti* following direct intrathoracic microinjection. At least five mosquitoes were tested for each of infections. At 5-day post infection, individual mosquitoes were homogenized using 1%NP40-PBS buffer, whereupon the supernatant was tested using the DENV serotype NS1 multiplex LFIA as well as RT-PCR based on RNA extracted from the same residual lysate. The results obtained using the NS1 multiplex LFIA in detecting and distinguishing mono-infections of DENV1~4 and co-infection with two serotypes were same as those results obtained *via* RT-PCR. Both of the methods detected all five mosquitoes infected with one serotype or two serotypes, as shown in **Table 9** and **Supplementary Figure 4**. The Dengue serotype NS1 multiplex LFIA did not produce any false positive results when tested against JEV, ZIKV, WNV, YFV, or CHIKV-infected mosquitoes (see **Table 9** and **Supplementary Figure 5**). Overall, the Dengue serotype NS1 multiplex LFIA performed very well in detecting NS1 proteins and differentiating DENV serotypes in individual infected mosquitoes, presenting no cross-reactivity with non-dengue virus in infected mosquitoes. Moreover, the Dengue serotype NS1 Multiplex LFIA proved highly effective in detecting double-infections and identifying the corresponding serotypes.

**Table 9:** Validation results for DENV serotype NS1 multiplex LFIA for mosquitoes infected with flaviviruses or Chikungunya virus.

Intrathoracic injection of virus in *Aedes aegypti*	N=	Test results using					
		Real-time-PCR	DENV serotype NS1 multiplex LIFA				
DENV mono-infection				D1	D2	D3	D4
DENV1	5	5/5 D1 positive	5/5	+	–	–	–
DENV2	5	5/5 D2 positive	5/5	–	+	–	–
DENV3	5	5/5 D3 positive	5/5	–	–	+	–
DENV4	5	5/5 D4 positive	5/5	–	–	–	+
**DENV co-infection**							
DENV1/DENV2	5	5/5 D1/D2 positive	5/5	+	+	–	–
DENV1/DENV3	5	5/5 D1/D3 positive	5/5	+	–	+	–
DENV1/DENV4	5	5/5 D1/D4 positive	5/5	+	–	–	+
DENV2/DENV3	5	5/5 D2/D3 positive	5/5	–	+	+	–
DENV2/DENV4	5	5/5 D2/D4 positive	5/5	–	+	–	+
DENV3/DENV4	5	5/5 D3/D4 positive	5/5	–	–	+	+
**Other flaviviruses**							
Zika virus	5	5/5 ZIKV positive	5/5	–	–	–	–
West Nile virus	5	5/5 West Nile positive	5/5	–	–	–	–
Japanese encephalitis virus	5	5/5 JEV positive	5/5	–	–	–	–
Yellow fever virus	5	5/5 YF positive	5/5	–	–	–	–
**Alphavirus**							
Chikungunya virus	5	5/5 CHIKV positive	5/5	–	–	–	–

## 4 Discussion

We previously developed an ELISA for the detection of dengue NS1 antigens and the differentiation of dengue serotypes in early-phase clinical serum samples. The assay involved pairing a serotype-cross-reactive monoclonal antibody (mAb) with one of four serotype-specific mAbs. In that study, we demonstrated that the selected DENV mAb pairs did not cross-react with ZIKV or JEV (39). In the current study, we performed a series of experiments with the goal of selecting mAb pairs for the assembly of the multiplex immunochromatographic format. Analysis was performed on four serotype-specific mAbs in 12 combinations with the aim of characterizing the system in terms of sensitivity (via serial dilution of supernatant from Vero cell cultures infected with DENVs) and specificity (ability to differentiate among the DENV serotypes). Finally, the optimal combination [stripe1 D1(T1), D4(T2), strip2 D2(T1), D3(T2)] presented minimal mutual interference with other test lines and background signals. The multiplex LFIA provides multi-target detection capability; however, the sensitivity and specificity of the proposed system can be affected by several factors in multi-parameter lateral flow detection. In other words, the results could be skewed by interference between multiple antigens and antibodies, differences in binding affinity among antibodies, and differences in testing procedures (44). The multiplex LFIA developed in this study eliminates the need for refrigeration and can be performed by non-laboratory personnel, thereby lowering costs and facilitating field surveillance and outbreak investigations. Furthermore, results from blood testing can be obtained in far less time than is required for RT-PCR (15 min versus 2-4 hours). The short turnaround time is expected to produce large benefits for clinical and public health intervention. In terms of analytic sensitivity, the limits of detection for each serotype were 31.25 ng/mL for DENV1, DENV2, and DENV4 and 15.625 ng/mL for DENV3 (**Table 2**). The limit of detection of multiplex LFIA unable to reach those sensitivities of dengue serotype NS1 ELISA (minimum detection levels of 1 - 4 ng/ml) (39), because the HRP conjugated antibodies used in ELISA amplify the signal to improve sensitivity. When using the same four purified NS1 proteins, the minimum detection levels of the commercial Bio-Rad Dengue NS1 Ag strip, which can’t differentiate dengue virus serotypes, were 15.6 ng/ml, 125 ng/ml, 15.6 ng/ml, and 61.5 ng/ml for DENV1 to DENV4, respectively. These results demonstrate the superior sensitivity of our multiplex LFIA for the detection of DENV NS1, while enabling the detection of various dengue virus serotypes at the same time. The efficacy of our multiplex LFIA was validated using culture supernatant from Vero cells infected with flaviviruses and chikungunya virus. We observed no cross-reactions with JEV, ZIKV, WNV, YFV, or chikungunya virus (**Tables 3** and **Supplementary Figure 3**). Chikungunya virus was selected for specificity testing because it frequently co-circulates with dengue in many dengue endemic regions. Sensitivity and accuracy in the detection of serotype were evaluated using acute phase of clinical serum samples from 187 patients presenting with fever, including 105 patients with confirmed dengue viral infections and 82 patients with non-dengue viral infections. The Dengue serotype NS1 Multiplex LFIA demonstrated high sensitivity to dengue virus in human clinical samples: D1 (90.0%), D2 (88.24%), D3 (82.61%), and D4 (83.33%) (**Table 7**). The multiplex LFIA also demonstrated high accuracy in the detection of serotype: D1 (97.35%), D2 (94.71%), D3 (97.33%), and D4 (95.72%) (**Table 7**). One of the clinical samples examined in this study presented negative results when analyzed using the serotype RT-PCR test, but positive results when using the dengue IgM/IgG test and DENV NS1 Ag ELISA for serotype. A secondary operator repeated the sample by the multiple LFIA also show positive D2 result. The multiplex LFIA identified this sample as positive for D2, the secreted NS1 protein amount and duration period is enough to detected by the multiplex LFIA, despite that dengue virus viremia was low in this serum sample and can’t be detected by RT-PCR. We assayed NS1 for the dengue serotype virus in laboratory-infected mosquitoes as a substitute by which to verify the efficacy of the multiplex LFIA in detecting dengue mono-infection and co-infections in endemic areas. When using our multiplex LFIA or molecular methods, all mosquitoes infected with mono/co dengue serotypes tested positive with no false positive results when tested against JEV, ZIKV, YFV, WNV, or CHIKV infections (**Table 9**, **Supplementary Figures 3** and **5**). Overall, the proposed multiplex LFIA proved highly effective in detecting DENV1-4 mono-infections and two serotype co-infections in individual mosquitoes (**Table 9** and **Supplementary Figure 4**). The proposed LFIA is able to detect instances of infection with multiple serotypes of dengue virus and identify the corresponding serotypes. The dengue serotype NS1 multiplex LFIA could be a valuable tool to provide quick and accurate dengue serotype diagnosis by which to ensure the administration of appropriate clinical management and facilitate the triaging of febrile patients under dengue outbreak conditions. The dengue serotype NS1 multiplex LFIA also provides a simple approach to identifying instances of co-infection involving more than two serotypes, which might otherwise complicate clinical treatment decisions. We did not observe cross-reactivity among serotypes, other flaviviruses, or CHIKV when using the multiplex LFIA to test NS1 proteins derived from the supernatant of cultured cells or mosquitoes infected with the virus. Nonetheless, we observed some cross-reactivity in a few of the clinical serum samples. This observed cross-reactivity of few blood samples might be caused from the background components of blood than that of cell cultures and mosquitoes.

One previous study reported on the use of paper-fluidic lateral immunoassays with four individual strips for DENV1-4 serotype detection (38). That test is able to distinguish among dengue virus serotypes with no cross-reactivity with Zika virus; however, it remains in the development stage. No commercially available NS1 ELISA kits or rapid lateral flow tests is able to distinguish serotypes or deal effectively with co-infections. Furthermore, some commercial dengue antigen diagnostic tests have produced false-positives in detecting the dengue virus NS1 antigen when applied to patients infected with Zika virus (45). Other tests have produced false-positives when applied to the culture supernatant of cells infected with other flaviviruses (46). False positives results can lead to overestimates of the burden associated with dengue (34).

**Figure 3:**
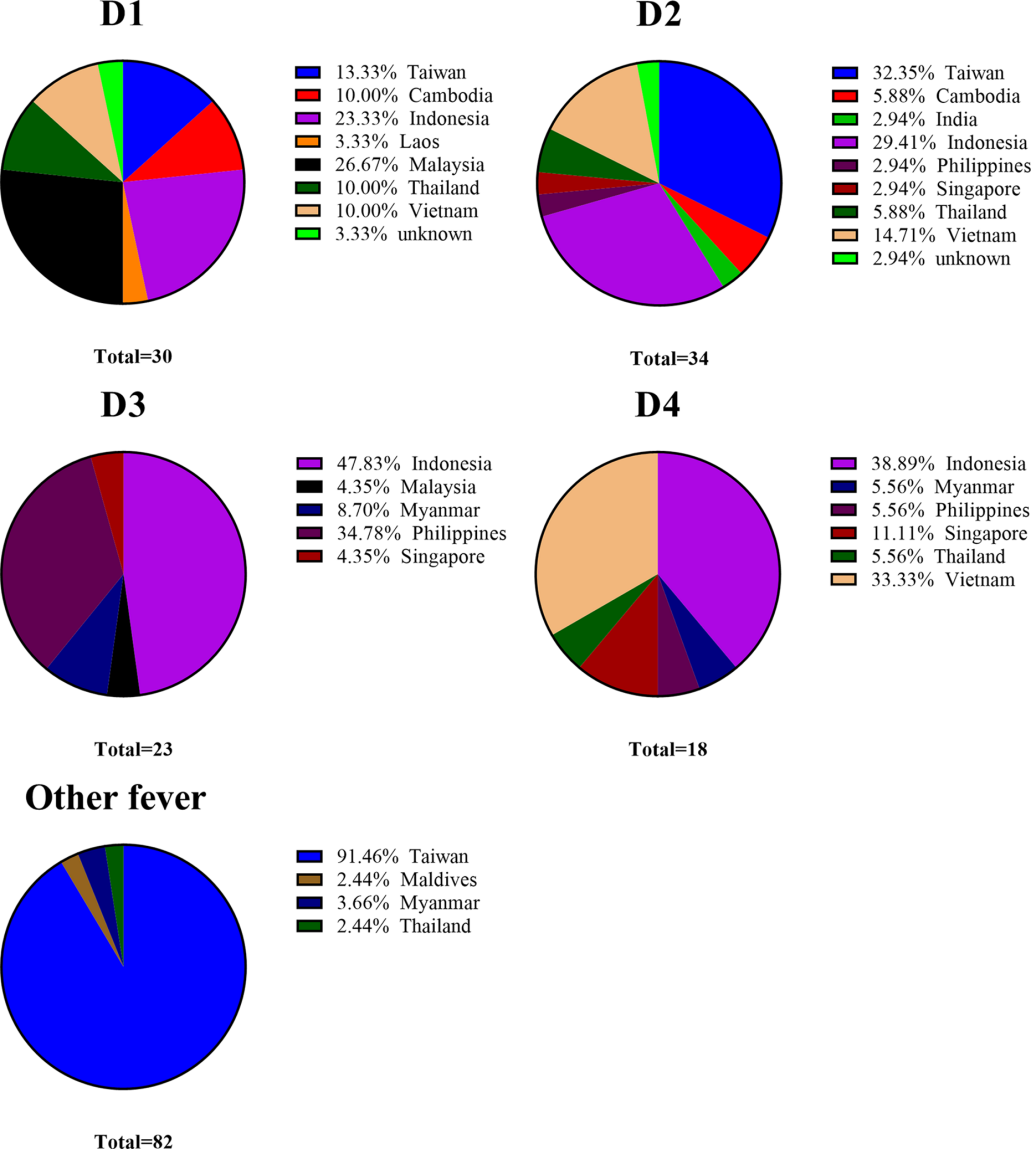
Distribution of DENV and other fever clinical serum samples collected from different regions.

Dengue fever is non-endemic in Taiwan; i.e., the indigenous form of dengue is the result of disease importation from dengue-endemic regions *via* commercial trade, travel, and human migration and following outbreak with local *Aedes* mosquitoes (47, 48). Taiwan has implemented entry screening at airports for the early detection of febrile passengers with dengue infection (49, 50). Suspected cases of dengue must be reported and specimens sent to surveillance authorities under Taiwan CDC for a clinical diagnosis within 24 hours (51). Overall, the cumulative number of dengue importations reported annually is positively correlated with the number of domestic cases (47). The vector mosquito *Aedes albopictus* is found throughout Taiwan, and *Aedes aegypti* is restricted to the southern part of the Island (51). In this study, 90 of the 105 dengue infected samples were imported from dengue-endemic regions in south and southeast Asia (Indonesia, Vietnam, Philippine, Malaysia, Thailand, Cambodia, Singapore, Myanmar, India, and Laos) between 2016 and 2020 (see **Table 5-1** and **Figure 3**). Those serum samples were obtained from the national surveillance system of the Taiwan Centers for Disease Control. Note that those samples covered a broad range of geographic regions, thereby demonstrating the efficacy of the Dengue serotype NS1 multiplex LFIA in the detection of dengue serotypes regardless of which genotypes in the circulating endemic regions.

In many countries, dengue is hyper-epidemic, with all four serotypes circulating simultaneously (52). Compared to cases of mono-infection, co-infections tend to result in more severe clinical manifestations (53, 54). At present, molecular testing is the only method capable of identifying dengue virus serotypes and detecting instances of multi-infection. However, molecular testing requires laboratory facilities with high diagnostic competence. There is a pressing need for a simpler detection method, such as antigen detection. At present, no commercial dengue antigen detection kit is able to identify dengue serotypes. The multiplex LFIA developed in this study provides a quick tool for the detection of co-infections and the identification of specific dengue serotypes.

The proposed Multiplex LFIA enables the real-time reporting of cases for clinical management, the field survey of serotypes, and the detection and characterization of co-infections. It can also be used to identify instances in which individuals who were previously infected by one serotype are subsequently infected by another serotype; i.e., patients under high risk of developing dengue hemorrhagic fever (16). It could also simple be used in the surveillance of DENV in field mosquito populations and/or to elucidate the dynamics of dengue infection in human or mosquito populations in endemic geographic regions.

This study was subject to a number of limitations. (1). Sample collection was limited by difficulties in obtaining dengue infected serum samples before symptom onset. (2). The dengue viral NS1 antigen can only be detected in the blood for a period of 9 days after the time of disease onset. This means that dengue NS1 antigen detection can only be used during the acute phase.

In summary, this study developed a multiple LFIA capable of rapidly detecting dengue virus NS1 antigens in early disease stages and identifying the specific serotype of dengue virus responsible for the infection. This could facilitate the rapid dissemination of information to health authorities and epidemiologists (to prevent transmission) and clinicians (to institute suitable management strategies aimed at reducing the incidence of severe cases). The proposed multiple LFIA is inexpensive, user-friendly, and ideally suited to use in dengue-endemic regions with limited laboratory facilities.

## Data Availability Statement

The original contributions presented in the study are included in the article/**Supplementary Material**. Further inquiries can be directed to the corresponding author.

## Ethics Statement

The studies involving human participants were reviewed and approved by the Kaohsiung Armed Forces General Hospital Institutional Review Board (IRB no. KAFGH 104-215 048). The patients/participants provided their written informed consent to participate in this study.

## Author Contributions

S-CL conceived and designed this study. C-CL supervised and reviewed the entire study. Y-YH: immunization of mice, hybridoma production, and mAb screening. S-CL and J-JW: (1) purification, characterization, and pairing of mAbs; (2) production and purification of DENV NS1 proteins; and (3) performed dengue NS1 capture ELISA for serotype. M-HT and Y-JH supervised this study. P-YS and S-FC: (1) performed dengue serotyping RT-PCR and dengue-specific IgM/IgG capture ELISA, and (2) provided confirmed clinical serum samples. Y-LC: (1) purification of mAbs and DENV NS1 proteins, and (2) molecular testing. J-NH manufactured the DENV serotype NS1 multiplex LFIA. C-CL performed intrathoracic inoculation of Aedes aegypti with DENV and molecular testing. S-CL and C-CL analyzed data, prepared figures and wrote the manuscript. All authors contributed to the article and have approved the submitted version.

## Funding

This work was supported by the following grants: 108-G3-2, 109-G3-2, and 110-G3-2 from the Institute of Preventive Medicine, National Defense Medical Center, Taiwan, Republic of China; NHRI-11A1-MRCO-08222201 and NHRI-110A1-MRCO-08212101 from the National Health Research Institute, Taiwan, Republic of China; and MOST 110-2327-B-016-002 from the Ministry of Science and Technology, Taiwan, Republic of China.

## Conflict of Interest

Author J-NH is employed by Trison Technology Corporation.

The remaining authors declare that the research was conducted in the absence of any commercial or financial relationships that could be construed as a potential conflict of interest.

## Publisher’s Note

All claims expressed in this article are solely those of the authors and do not necessarily represent those of their affiliated organizations, or those of the publisher, the editors and the reviewers. Any product that may be evaluated in this article, or claim that may be made by its manufacturer, is not guaranteed or endorsed by the publisher.
