# Supplementary material for: Development of Novel Dengue NS1 Multiplex Lateral Flow Immunoassay to Differentiate Serotypes in Serum of Acute Phase Patients and Infected Mosquitoes
Source: Front Immunol. 2022 Mar 4;13:852452. doi: 10.3389/fimmu.2022.852452 (PMC8931297; doi:10.3389/fimmu.2022.852452)
Supplement: Supplementary file 1 [file DataSheet_1.pdf]

Supplementary Material

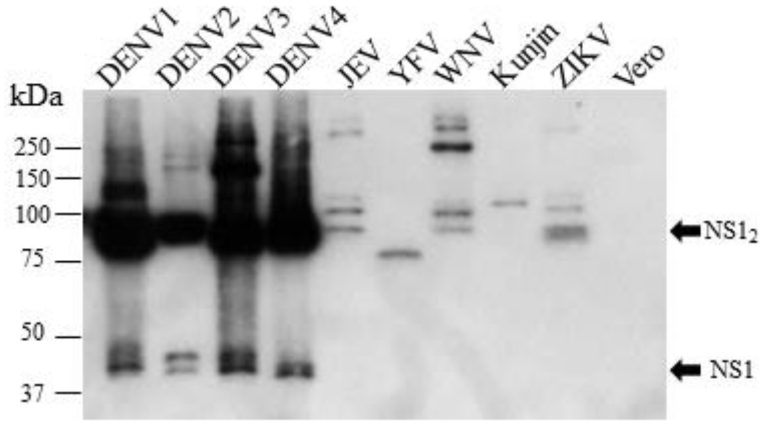

Supplementary Figure 1. Detection of NS1 antigen from the various flaviviruses in the supernatant of infected Vero cells using monoclonal antibody D_2 8-1 (flavivirus-group) against NS1 proteins.

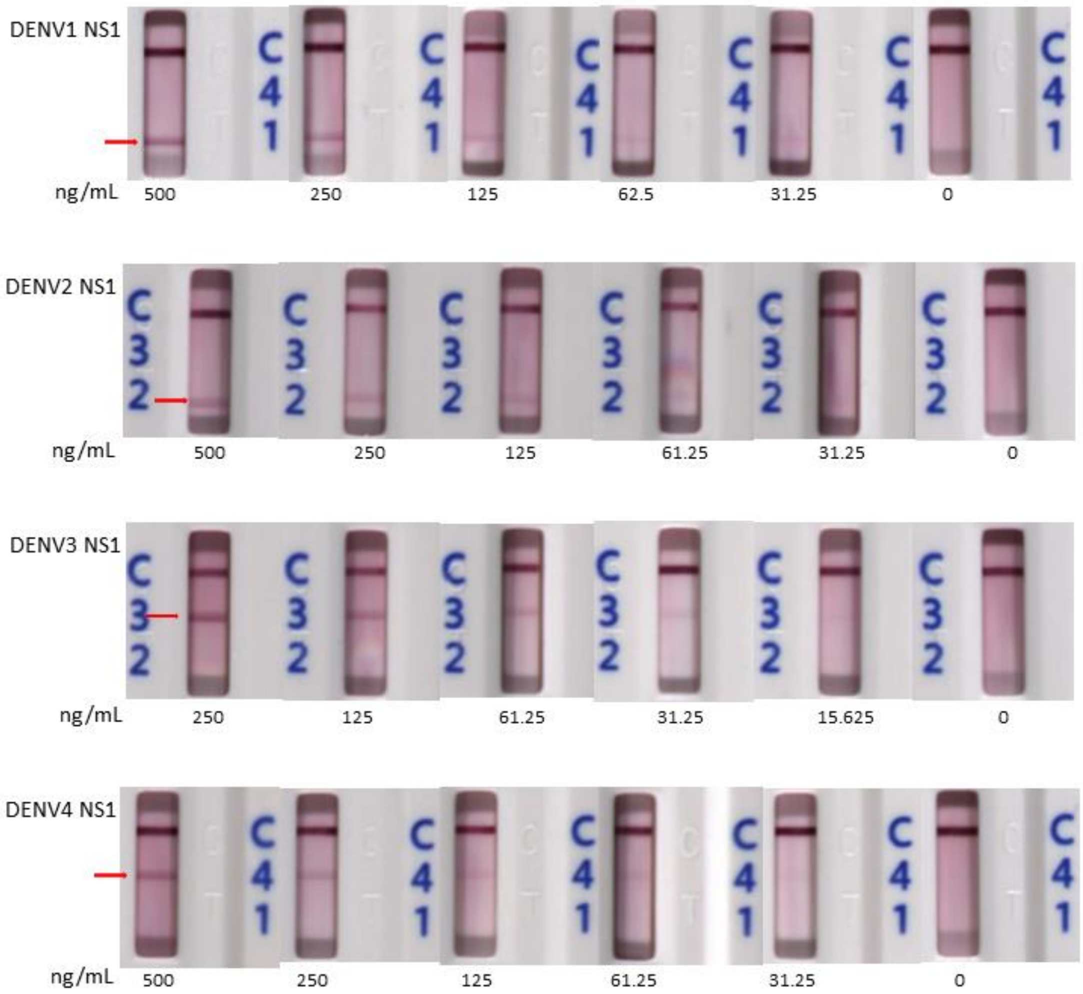

Supplementary Figure 2. Detection limits of the proposed DENV serotype NS1 multiplex LFIA chromatographic detection system using solutions containing immunoaffinity-purified DENV NS1 proteins. The limit of detection of NS1 multiplex LFIA was 31.25 ng/mL for DENV1, 31.25 ng/mL for DENV2, 15.625 ng/mL for DENV3, and 31.25 ng/mL for DENV4.

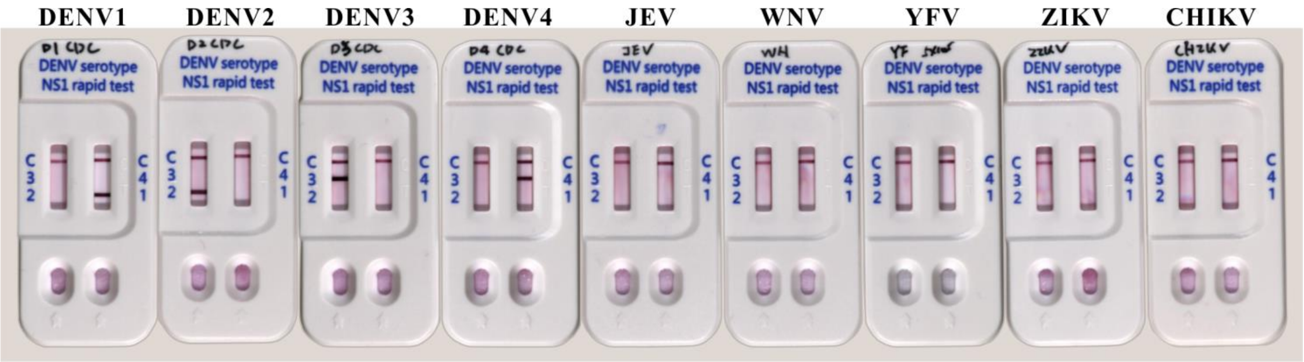

Supplementary Figure 3. Cross-reactivity of DENV serotype NS1 multiplex LFIA. Photographs showing DENV serotype NS1 multiplex LFIA using cell culture supernatant from Vero cells infected with DENV1, 2, 3, or 4 or JEV, ZIKV, WNV, YFV, or CHIKV.

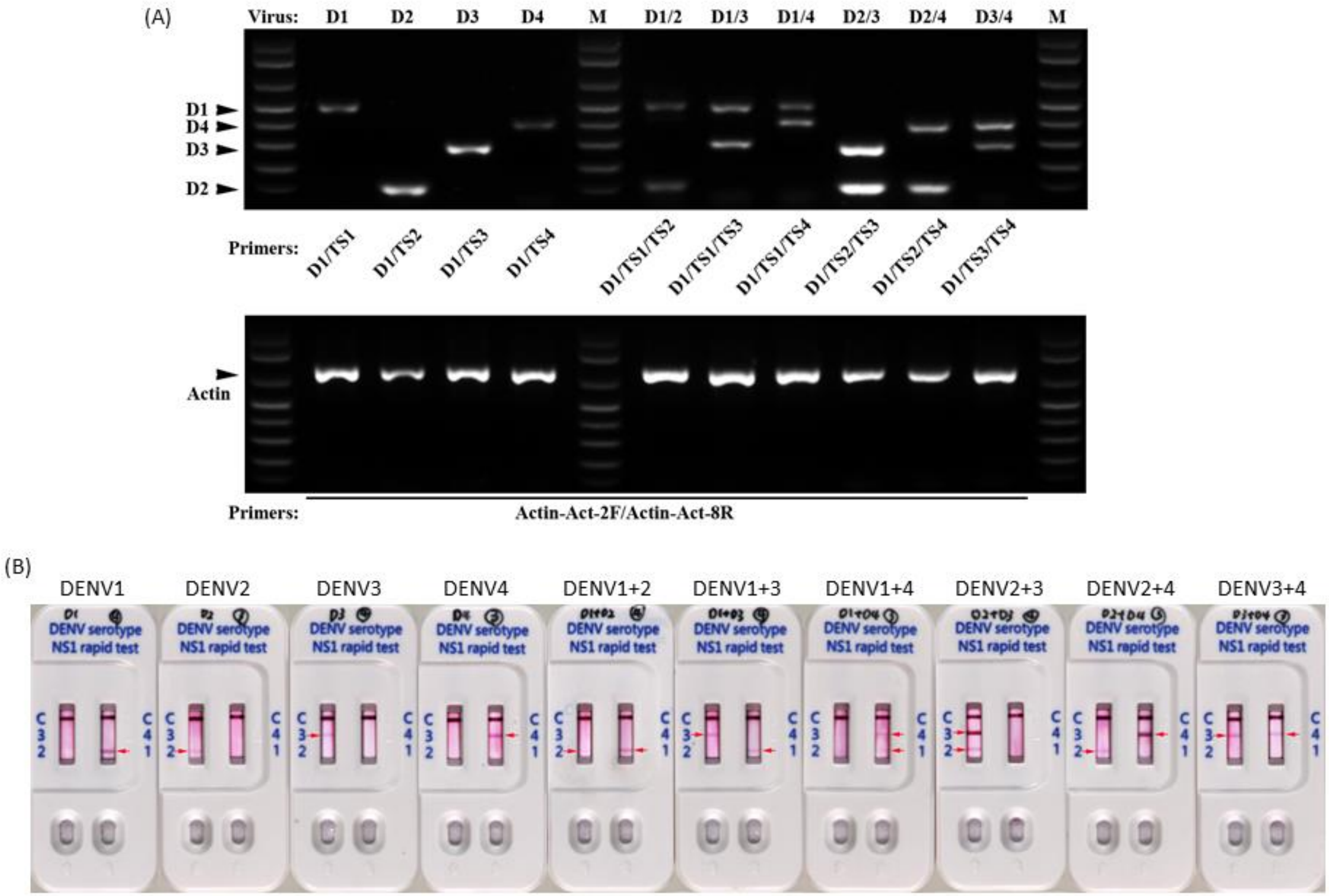

Supplementary Figure 4. Samples of individual dengue virus mono-infection (D1, D2, D3, and D4) and co-infection (D1+D2, D1+D3, D1+D4, D2+D3, D2+D4, and D3+D4) mosquitoes tested using dengue serotype-specific RT-PCR and the proposed DENV serotype NS1 multiplex LFIA. (A) RNA reversed transcripted into cDNA from dengue virus mono-infected and co-infected mosquitoes was subjected to PCR using dengue serotype specific primers with actin as an internal control. DENV1 amplicon size: 482bp, DENV2 amplicon size: 119bp, DENV3 amplicon size: 290bp, DENV4 amplicon size: 392bp, and actin amplicon size: 660bp. The amplification PCR products were analyzed using 1% agarose gel electrophoresis with ethidium bromide. (B) The same individual virus infected-mosquitoes were homogenized with 1%NP40-PBS buffer, after which the supernatants were tested using the proposed DENV serotype NS1 multiplex LFIA. The results were read by naked eye at 15 min.

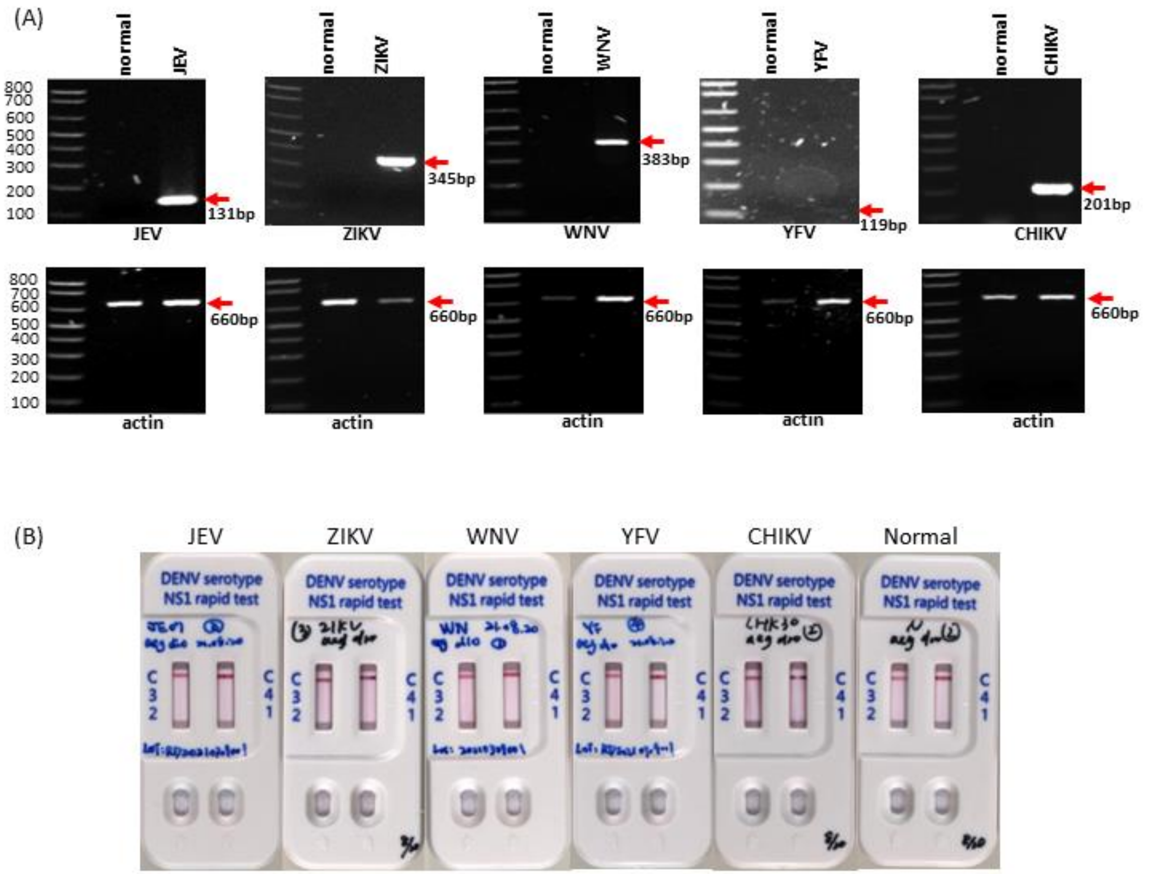

Supplementary Figure 5. Samples of individual JEV, ZIKV, YFV, and CHIKV infected mosquitoes tested using specific PCR and the proposed DENV serotype NS1 multiplex LFIA. (A) RNA Reversed transcipted into cDNA from JEV-, ZIKV-, YFV-, and CHIKV-infected individual mosquitoes was subjected to PCR using specific primers and with actin as an internal control. YFV amplicon size: 119bp, WNV amplicon size: 383bp, JEV amplicon size: 131bp, ZIKV amplicon size: 345bp, CHIKV amplicon size: 201bp, and actin amplicon size: 660bp. The amplification PCR products were analyzed using 1% agarose gel electrophoresis with ethidium bromide (B). The same individual virus infected-mosquitoes were homogenized with 1%NP40-PBS buffer, whereupon the supernatants were tested using the proposed DENV serotype NS1 multiplex LFIA. The results were read by the naked eye at 15 mi.

Supplementary Table 1. Primer sequences for dengue virus serotype-specific RT-PCR

Primers	Sequence (5’ to 3’)	Serotype	Genomic region	PCR Size (bp)	Reference
D1	5'-TCAATATGCTGAAACGCGCGAGAAACCG-3'	DENV (all)	134-161		(1)
TS1	5'-CGTCTCAGTGATCCGGGGG-3'	DENV1: D1- TS1	568-586	482	
TS2	5'-CGCCACAAGGGCCATGAACAG-3'	DENV2: D1- TS2	232-252	119	
TS3	5'-TAACATCATCATGAGACAGAGC-3'	DENV3: D1- TS3	400-421	290	
TS4	5'-CTCTGTTGTCTTAAACAAGAGA-3'	DENV4: D1- TS4	506-527	392	

Supplementary Table 2. Primer sequences for flaviviruses and Chikungunya virus RT-PCR

Primers	Sequence (5’ to 3’)	GeneBank reference no.	Genomic region	PCR Size (bp).	Reference
YF-V-F	GTATTCTGTGGATGCTGACC	NC 002031	10312-10331	119	(2)
YF-V-R	TATCCCGGTTTCAGGTTGTG		10412-10431		
WN-F	TYGTGTTGGCTCTYTTGGCGTTYTT	AY532665	233-257	383	(3)
WN-R	CAGCMGMCAGSACTGGACAYTCATA		640-616		
JE-5941	GAAACCCATCTCCCATAACC	JEV/Taiwan/TP0605a/M/2005 (KF667310)	5914-5933	131	In house primer
JE-6053	AATGGGCTAGGTTACTGTCA		6026-6045		
ZIKA-ENV-F*	GCTGGDGCRGACACHGGRACT (2008, original) GCTGGGGCAGACACCGGAACT(2019,modified)	AY532665	1538-1558	345	(4)
ZIKA-ENV-R*	RTCYACYGCCATYTGGRCTG (2008, original) GTCCACGCCATCTGAGCTG (2019,modified)		1902-1883		
CHIKV-CK2	GTCTGTTCTACACAAGTACAC	FJ807897	11086-11106	201	(5)
CHIKV-CK3	ACGACACGCATAGCACCAC		11269-11287		
actin-Act-2F	ATGGTCGGYATGGGNCAGAAGGACTC	U02933.1	269-294	660	(6)
actin-Act-8R	GATTCCATACCCAGGAAGGADGG		951-929		

*We modified the primer of ZIKV from original established in J clin Microbiol 2008 43(1):96-101.

Supplementary Reference

1. Chien L-J, Liao T-L, Shu P-Y, Huang J-H, Gubler DJ, Chang G-JJ. Development of real-time reverse transcriptase PCR assays to detect and serotype dengue viruses. J Clin Microbiol (2006) 44(4):1295-304. doi: 10.1128/JCM.44.4.1295-1304.2006
2. Hughes HR, Russell BJ, Mossel EC, Kayiwa J, Lutwama J, Lambert AJ. Development of a Real-Time Reverse Transcription-PCR Assay for Global Differentiation of Yellow Fever Virus Vaccine-Related Adverse Events from Natural Infections. J Clin Microbiol (2018) 56(6). doi: 10.1128/JCM.00323-18
3. Lanciotti RS, Kerst AJ, Nasci RS, Godsey MS, Mitchell CJ, Savage HM, et al. Rapid detection of west nile virus from human clinical specimens, field-collected mosquitoes, and avian samples by a TaqMan reverse transcriptase-PCR assay. J Clin Microbiol (2000) 38(11):4066-71. doi: 10.1128/JCM.38.11.4066-4071.2000
4. Faye O, Faye O, Dupressoir A, Weidmann M, Ndiaye M, Alpha Sall A. One-step RT-PCR for detection of Zika virus. J Clin Virol (2008) 43(1):96-101. doi: 10.1016/j.jcv.2008.05.005
5. Ho Y-J, Wang Y-M, Lu J-w, Wu T-Y, Lin L-I, Kuo S-C, et al. Suramin Inhibits Chikungunya Virus Entry and Transmission. PLOS One (2015) 10(7):e0133511. doi: 10.1371/journal.pone.0133511
6. Staley M, Dorman KS, Bartholomay LC, Fernández-Salas I, Farfan-Ale JA, Loroño-Pino MA, et al. Universal primers for the amplification and sequence analysis of actin-1 from diverse mosquito species. J Am Mosq Control Assoc (2010) 26(2):214-8. doi: 10.2987/09-5968.1
